# Supercritical CO_2_ Assisted Impregnation of Ibuprofen on Medium-Chain-Length Polyhydroxyalkanoates (mcl-PHA)

**DOI:** 10.3390/molecules26164772

**Published:** 2021-08-06

**Authors:** Liane Meneses, Rita Craveiro, Ana Rita Jesus, Maria A. M. Reis, Filomena Freitas, Alexandre Paiva

**Affiliations:** 1 LAQV-REQUIMTE, Chemistry Department, NOVA School of Science and Technology, NOVA University of Lisbon, 2825-149 Caparica, Portugal; lp.meneses@campus.fct.unl.pt (L.M.); rita.craveiro@campus.fct.unl.pt (R.C.); ar.gameiro@fct.unl.pt (A.R.J.); alexandre.paiva@fct.unl.pt (A.P.); 2 Associate Laboratory i4HB—Institute for Health and Bioeconomy, School of Science and Technology, NOVA University Lisbon, 2815-149 Caparica, Portugal; amr@fct.unl.pt; 3 UCIBIO—Applied Molecular Biosciences Unit, Department of Chemistry, School of Science and Technology, NOVA University Lisbon, 2825-149 Caparica, Portugal

**Keywords:** medium-chain-length polyhydroxyalkanoates, supercritical carbon dioxide, ibuprofen, impregnation, green solvents, alternative technologies, controlled drug release

## Abstract

In this work, we propose the utilization of scCO_2_ to impregnate ibuprofen into the mcl-PHA matrix produced by *Pseudomonas chlororaphis* subs. *aurantiaca* (DSM 19603). The biopolymer has adhesive properties, is biocompatible and has a melting temperature of 45 °C. Several conditions, namely, pressure (15 and 20 MPa) and impregnation time (30 min, 1 h and 3 h) were tested. The highest ibuprofen content (90.8 ± 6.5 mg of ibuprofen/g_PHA_) was obtained at 20 MPa and 40 °C, for 1 h, with an impregnation rate of 89 mg/(g·h). The processed mcl-PHA samples suffered a plasticization, as shown by the decrease of 6.5 °C in the T_g_, at 20 MPa. The polymer’s crystallinity was also affected concomitantly with the matrices’ ibuprofen content. For all the impregnation conditions tested the release of ibuprofen from the biopolymer followed a type II release profile. This study has demonstrated that the mcl-PHA produced by *P. chlororaphis* has a great potential for the development of novel topical drug delivery systems.

## 1. Introduction

Drug delivery systems (DDS) are of extreme importance in the medical field because they allow the delivery of a balanced drug concentration, the control of drug administration over long periods of time and the protection of bioactive compounds that have a short half-life. DDS also reduce the drugs’ side effects, drug wastage and over medicating, and contribute to treatment optimization and to patient compliance [1]. The preparation of DDS usually comprises three steps: (1) solubilization of the active pharmaceutical ingredient (API) in an appropriate solvent; (2) diffusion of the API into the polymeric matrix and, finally, (3) removal of the solvent [2].

Supercritical CO_2_ (scCO_2_)-assisted impregnation has been used for the development of several DDS due to ability of scCO_2_ to easily diffuse into a variety of polymeric matrices, causing their swelling, thus increasing the structures’ free volume [2]. ScCO_2_ is also capable of solubilizing a variety of APIs, including anti-inflammatory drugs, and carrying them into the polymeric matrices [3]. This is especially interesting when working with APIs that are thermosensitive (labile), since the critical point of CO_2_ is at relatively mild conditions (31 °C, 7.38 MPa) [3]. Furthermore, scCO_2_ has a low environmental impact: it is inert and non-flammable and easy to remove from the polymer, thus resulting in a product with no solvent residues [3,4]. Processing polymers with scCO_2_ has other advantages, including the possibility of altering their mechanical and physical properties. Sorption of scCO_2_ into polymers can lead to their plasticization, in which a reduction of the glass transition temperature (T_g_) is observed [5]. Furthermore, the application of such alternative solvents also allows for the production of functional porous materials [6]. The scCO_2_-assisted impregnation of non-inflammatory drugs has been applied for the fabrication of several biomaterials, such as intraocular [7] and contact lenses [8], urethral stents [9], aerogel microspheres [10], chitosan films for oral mucosal drug delivery [11], scaffolds for tissue engineering [12] and even in mesoporous nanostructured ZnO [13]. ScCO_2_ impregnation has also been reported for the production of polycaprolactone patches impregnated with nimesulide [14].

Polyhydroxyalkanoates (PHAs) are biobased, biodegradable and biocompatible plastics [15]. These characteristics make them suitable candidates for use in medical applications, such as DDS. Medium-chain-length PHAs (mcl-PHAs) are composed of monomers with six to fourteen carbon atoms and are characterized by their low melting (40 to 60 °C) and glass transition (−50 to −25 °C) temperatures, low crystallinity degrees (<40%), low tensile strength (maximum 10 mPa) and high elongation at break ratios (c.a. 300%) [16]. The intrinsic properties of mcl-PHAs give these biopolymers glue-like behavior and adhesive properties that potentiate their application in the biomedical field as patches, rubbers or glues [16,17,18,19]. Recently, the potential of the mcl-PHA produced by *Pseudomonas chlororaphis* subs. *aurantiaca* (DSM 19603) to be used as a skin adhesive was demonstrated [19]. Upon impregnation with APIs—either antibiotics, anti-inflammatory drugs or anti-cancer drugs—such adhesives could also be used as DDS [20].

Over the last few decades, PHAs have been intensively investigated for the development of several biomedical applications. For instance, scaffolds resulting from the combination of mcl-PHA and poly(3-hydroxybutyrate) (P(3HB)) were shown to effectively promote the proliferation of different human cell lines [21]. A mcl-PHA-based transdermal DDS has also been described for the successful delivery of tamsulosin in a skin model [22]. Other PHAs, namely P(3HB) and poly(3-hydroxybutyrate-co-3-hydroxyvalerate) (P(HB-*co*-HV)), have been used in tissue engineering as bone rods, prodrugs, tablets or micro-carriers [20,23,24]. For their application as bone rods, P(HB-*co*-HV) was loaded with antibiotics and applied for the treatment of osteomyelitis. For this purpose, the rods were prepared by the solvent casting method [23,24]. P(3HB) has also been used for the preparation of tablets, in which the API and the biopolymer were mixed as powders and then compressed [20]. The impregnation methods used in such studies carry a series of disadvantages, namely the impact of the applied mechanical and thermal conditions on the therapeutic effect of the drugs, or the use of organic solvents such as chloroform that must be completely removed from the impregnated matrices [6,25]. The processing of P(HB-*co*-HV) and P(3HB) with scCO_2_ has already been reported [26,27]. In one work, the authors determined the solubility and diffusion coefficients of scCO_2_ into the biopolymer P(HB-*co*-HV), as well as the influence of the 3-hydroxyvalerate (3HV) content of the polymer on the CO_2_ sorption [26]. In a different study, the authors observed that upon scCO_2_ treatment of several biopolymers, including P(HB-*co*-HV) and P(3HB), there was a decrease in their melting temperature without any degradation of the polymer [27].

In this work we describe, for the first time, the scCO_2_-assisted impregnation of ibuprofen in the mcl-PHA produced by *Pseudomonas chlororaphis* subs. *aurantiaca* (DSM 19603.) Different impregnation conditions, namely pressure and time, were tested and their effect on impregnation yield was accessed. Furthermore, the biopolymer’s physical-chemical characteristics were evaluated to assess the impact of the tested impregnation conditions. The cytotoxicity of the biopolymer was also evaluated on two different cell lines to confirm its biocompatibility.

## 2. Results and Discussion

### 2.1. Characterization of the mcl-PHA

The biopolymer used in this study was a mcl-PHA composed of 3 wt% 3-hydroxyhexanoate (3HHx), 17 wt% 3-hydroxyoctanoate (3HO), 50 wt% 3-hydroxydecanoate (3HD), 13 wt% 3-hydroxydodecanoate (3HDd) and 17 wt% 3-hydroxytetradecanoate (3HTd) [28]. It had a high molecular weight (Mw) of 1.25 × 10^5^ g/mol with a low polydispersity index (PDI) of 1.45, which shows the homogeneity of the polymer macromolecular chains. The mcl-PHA presented a melting temperature (T_m_) of 45 °C and a glass transition temperature (T_g_) of c.a. −50 °C. Although its T_m_ is relatively low, the polymer’s degradation temperature (T_deg_) is of 288 °C, which offers a wide range of working temperatures at which the polymer can be processed. The biopolymer is a highly amorphous material, with a degree of crystallization (X_c_) of 27% [28]. This material was chosen for this impregnation study due to its low T_m_ (45 °C) that renders its impregnation by scCO_2_ easy to achieve.

**Figure 1:**
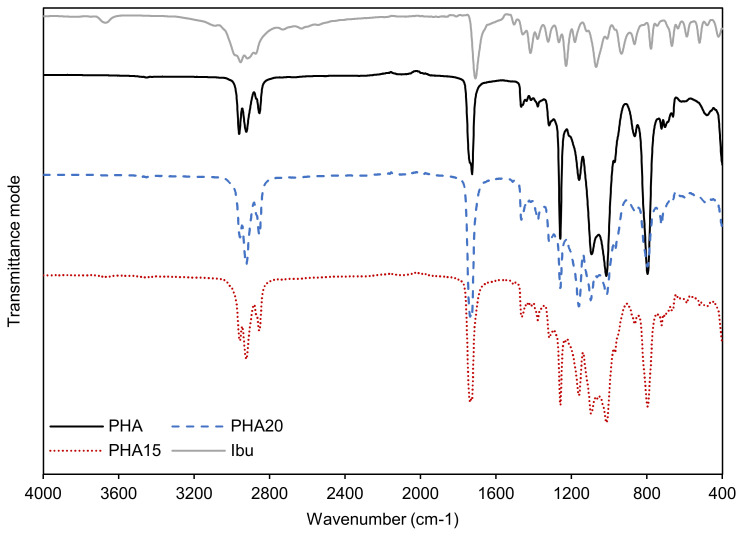
ATR-FTIR spectra of mcl-PHA original sample (PHA, black full line) compared to the mcl-PHA sample impregnated with ibuprofen at 20 MPa (PHA20, blue dashed line) and the sample impregnated at 15 MPa (PHA15, red dotted line). The spectrum for ibuprofen (Ibu, grey full line) is also represented. The spectra were obtained in the region of 4000 to 400 cm^−1^, in transmittance mode.

The ATR-FTIR spectrum of the mcl-PHA (Figure 1) displays the typical bands for PHAs [29,30]. Three defined bands are visible at 2962, 2921 and 2854 cm^−1^, which correspond to the methyl (-CH_3_) and methylene (-CH_2_) groups of the PHA molecule. The bands in this region are more intense for mcl-PHA than scl-PHA and are considered characteristic of mcl-PHA [31]. The band located at 1727 cm^−1^, corresponding to the stretching of the ester carbonyl (C=O) groups, is associated with the crystalline phase of the PHA and is also considered characteristic of PHAs [31,32]. The bands appearing between 1500–1000 cm^−1^ are assigned to stretching vibrations of the C-O bonds and have correlation with the degree of crystallinity; and finally, the bands appearing at 1015, 865 and 796 cm^−1^ represent C-C stretching and are also characteristic of PHA [30].

### 2.2. Evaluation of mcl-PHA Citotoxicity

**Figure 2:**
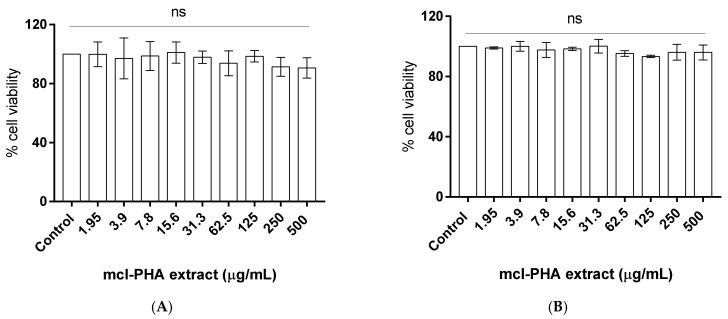
Cytotoxicity evaluation of mcl-PHA extract in (**A**) L929 cells and (**B**) HaCaT cells. Data represent means ± SD (*n* = 3). Statistically significant differences were determined by Dunnett’s multiple comparisons test, one-way ANOVA. There were no significant differences between the test groups and the control group.

Cell viability after 24 h of incubation with increasing concentrations of the mcl-PHA extract was evaluated using the MTS assay, in which the production of formazan is proportional to the cells’ viability. No loss of L929 (Figure 2A) or HaCaT (Figure 2B) cell viability was noticed up to 500 µg/mL. Considering that a cytotoxic effect is considered when cell viability decreases below 70%, according to ISO 10993-5 [33], these results show that the mcl-PHA produced by *P. chlororaphis* can be considered a safe biopolymer for further biological studies. The results obtained are in accordance with others found in literature. For instance, the toxicity of the homopolymer poly-(3)-hydroxyoctanoate, P(3HO), was evaluated on L929 murine fibroblast cells, and the results showed that P(3HO) not only presents no toxicity towards that cell line, but it also even promotes cell growth and adherence [34]. There are also studies on the effect of poly(3-hydroxybutyrate-co-3-hydrovalerate-co-3-hydroxyhexanoate (P(HB-*co*-HV-*co*-HHx) on the HaCaT cell line that have shown that the presence of the co-polymer promotes cell proliferation [35]. Besides the studies on PHA cytotoxicity, the toxicity of PHA degradation products has also been studied. The authors observed that the degradation products of mcl-PHA show less cytotoxicity towards mouse fibroblasts, hence suggesting that mcl-PHA are preferred for biomedical applications [36].

### 2.3. scCO_2_ Impregantion of mcl-PHA with Ibuprofen

Considering the mcl-PHA’s T_m_ (45 °C) [28], a temperature of 40 °C was chosen to conduct the impregnation assays, thus guaranteeing that the biopolymer was solid at room pressure but melted at the applied tested pressure values (15 and 20 MPa). The choice of these pressure values relied mostly on the solubility of ibuprofen in scCO_2_ [37]. Also, in previous works, these pressures proved to be the most effective for the impregnation of ibuprofen on polymeric matrices [14,38]. Complete mcl-PHA melting at this temperature was possible since at high pressures, scCO_2_ causes a melting point depression due to its solubilization in the polymer, as demonstrated for P(3HB) and P(HB-co-HV) [27]. Rendering the mcl-PHA in a liquid state combined with the low viscosity of supercritical fluids [39] facilitates the mass transfer of the scCO_2_ within the biopolymer matrix, hence resulting in a homogeneous impregnation of ibuprofen.

The impregnation of ibuprofen into the mcl-PHA matrices was tested at pressure values of 15 MPa for 3 h, and 20 MPa for 30 min, 1 h and 3 h (Table 1). The ibuprofen and mcl-PHA were both placed in a high-pressure vessel that was closed and placed in a water bath at 40 °C to assure that the temperature was stable. After each impregnation period, the system was slowly depressurized to guarantee complete CO_2_ removal from the polymer matrix, and the consequent ibuprofen precipitation that occurs at a constant rate.

**Figure 3:**
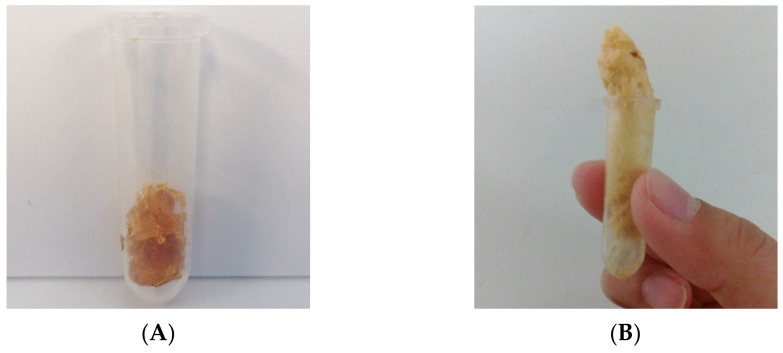
mcl-PHA before (**A**) and after (**B**) impregnation for 3 h at 20 MPa.

The sorption of scCO_2_ into the biopolymer matrix upon depressurization is clear and evidenced by its expansion (Figure 3B) compared to the original mcl-PHA sample (Figure 3A). This shows that exposure to the scCO_2_ treatment causes foaming of the biopolymer matrix, increasing its porosity and changing its morphology. The original mcl-PHA sample presented a glue-like behavior, associated to its highly amorphous structure (X_c_ = 27% and T_g_ = −50 °C) [19,28], which usually corresponds to a higher affinity towards CO_2_ than polymers with higher crystallinity degrees or high T_g_ values [40]. Sorption of CO_2_ into the polymeric matrix can cause swelling and plasticization, characterized by the reduction of the T_g_, as well as decrease of viscosity and improvement of the dispersion of additives through the biopolymer matrix during impregnation [5]. ScCO_2_ swelling and plasticization also increase the mobility of the polymer’s chains, hence turning glassy polymers into rubbery matrixes which allows for the generation of porous structures [39,41]. CO_2_ is also able to interact with polymers’ ester groups, hence causing enhanced mobility of the polymeric chains [5].

Impregnation of ibuprofen into the mcl-PHA matrix was confirmed by performing ATR-FTIR. Figure 1 shows the spectra of the two impregnated samples and pure ibuprofen. The ibuprofen spectrum (Ibu, in Figure 1) shows a well-defined band at wavenumber 1709 cm^−1^ that is related to C=O stretching. In the region from 3000 to 2600 cm^−1^ there is also a larger band with small peaks, which are associated with CH_3_ and CH_2_ asymmetric stretching [42]. As referred to above, the mcl-PHA has characteristic bands at the same wavenumbers. Comparing the spectra of the impregnated samples with that of the original mcl-PHA, an increase in intensity of those bands is observed. Small shifts from the characteristic C=O PHA band from 1727 cm^−1^ to 1738 cm^−1^ in both impregnated samples (PHA15 and PHA20) is also seen. Both these findings confirm the presence of ibuprofen within the mcl-PHA matrix. For the experiments conducted for 3 h at 15 and 20 MPa, ibuprofen contents of 66.7 ± 0.7 and 93.3 ± 4.7 mg/g_PHA_ were obtained, respectively (Table 1). These results showed that the pressure applied during the scCO_2_-assisted impregnation had a clear impact on the amount of ibuprofen that scCO_2_ was able to solubilize, hence determining the amount of ibuprofen loaded into the biopolymer’s matrix. This is explained by the fact that ibuprofen’s solubility in scCO_2_ increases with pressure [37,43]. Afterwards, two more impregnation times —30 min and 1 h —were studied, using a fixed pressure of 20 MPa to evaluate whether the shorter times would result in ibuprofen contents similar to the value obtained at 3 h. Since the impregnation of ibuprofen in the polymer is controlled by the diffusion rate of CO_2_ into the polymer matrix, it was expected that impregnation time would influence the amount of ibuprofen impregnated into the mcl-PHA. A similar ibuprofen content was reached for the experiment conducted for 1 h (90.8 ± 6.5 mg/g_PHA_) but further reducing the impregnation time to 30 min resulted in a significant reduction (40.9 ± 0.8 mg/g_PHA_) (Table 1). Therefore, comparing the results from the three experiments conducted at 20 MPa, it can be observed that the maximum ibuprofen concentration was obtained after 3 h of impregnation, but the value is similar to that obtained at 1 h, considering the standard deviation of both values.

There are few references of the loading of ibuprofen into PHAs. For instance, the loading of ibuprofen into P(3HB) microspheres has been performed using an evaporation method with dichloromethane. The authors were able to achieve a drug concentration in the P(3HB) microspheres of c.a. 160 mg/g_PHA_ [44]. Although this value is higher than the one reported in this study, the impregnation of APIs using organic solvents always carries disadvantages, such as the presence of residual amounts of solvent, which can be harmful. Besides this, this process of impregnation with scCO_2_ in mcl-PHA is still to be further optimized, hence allowing to increase the amount of ibuprofen in the biopolymeric matrix.

**Figure 4:**
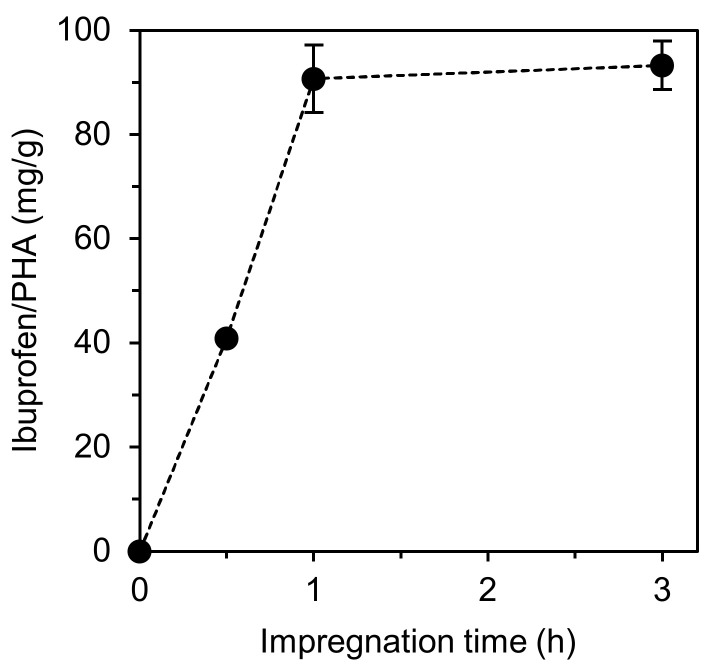
Maximum ibuprofen concentration on mcl-PHA vs. impregnation time for the experiments conducted at 20 MPa and 40 °C.

By plotting the maximum ibuprofen concentration against the corresponding impregnation time for the experiments conducted at 20 MPa, it was possible to determine the ibuprofen-impregnation rate into the mcl-PHA at that fixed pressure, which was determined as 89 mg/(g·h) (Figure 4). Here, it is also possible to observe that the maximum amount of ibuprofen that the PHA is capable of accommodating is achieved after only 1 h. If compared with the impregnation of ibuprofen on alginates, for instance, the maximum ibuprofen concentration (148 mg/g_Alginate_) was only achieved after 3 h of impregnation [2], representing a much slower impregnation rate than the one obtained in this work.

### 2.4. Characterization of the mcl-PHA Samples Impregnated with Ibuprofen

The degree of cristallinity (X_c_) of the PHA after scCO_2_-assisted impregnation was determined for the samples prepared for 3 h, at 15 and 20 MPa. These values differ from the X_c_ of the original mcl-PHA, 27% [28] and reflect the changes suffered by the biopolymer matrix upon scCO_2_ impregnation. On the one hand, a decrease of X_c_ was expected, due to the amorphization of the polymer caused by the sorption of CO_2_. On the other hand, due to the impregnation of ibuprofen, which is crystalline with a diffraction pattern showing a high density of peaks within 6.1° and 22.3° (2θ) [45], an increase of X_c_ may occur. For the sample impregnated with ibuprofen at 15 MPa, there was a decrease of c.a. 15% on the X_c_, while for the PHA20, the X_c_ increased c.a 22%. It is possible to conclude that for the PHA15, which had a lower ibuprofen loading, amorphization of the polymer upon CO_2_ sorption has a higher influence on crystallinity. Ibuprofen loading in the PHA20 was 1.4-fold higher, which may be responsible for the 22% increase in crystallinity. The effect of CO_2_ sorption in the polymer can be better understood by the decrease of T_g_. For PHA15 there was a decrease in the T_g_ of 1 °C, compared to the pure PHA, and for PHA20 a decrease of 6.5 °C was observed. This shows that CO_2_ has some plasticizing effect of the polymer.

### 2.5. Ibuprofen Release Studies

**Figure 5:**
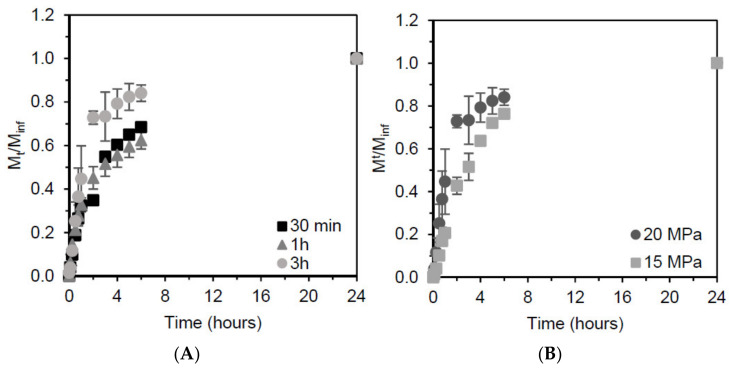
Release profile of ibuprofen from (**A**) mcl-PHA samples impregnated for different time periods (30 min, 1 h and 2 h) at 20 MPa and (**B**) mcl-PHA samples impregnated at 15 and 20 MPa for a period of 3 h.

Table 1 shows the initial release rate of ibuprofen in PBS, as well as the percentage of ibuprofen released to the medium during the first hour. From the ibuprofen release profiles (Figure 5) it is possible to see that the ibuprofen release over the first hour was faster than during the following 23 h. For the sample resulting from impregnation conducted for 3 h at 15 MPa, the initial ibuprofen release rate was 3.7 mg/h and 21% of the loaded ibuprofen was released from the mcl-PHA matrix within the first hour (Table 1). On the other hand, for the experiment conducted for the same time, but at 20 MPa, the initial ibuprofen release rate was 14.7 mg/h and with a release of 45% of ibuprofen released during the first hour (Table 1). For the other two experiments conducted at 20 MPa (30 min and 1 h), the initial ibuprofen release rates were 3.0 and 5.6 mg/h, respectively. For both these assays the ibuprofen released during the first hour accounted for c.a. 30% of the total amount of ibuprofen released during the 24 h studied (Table 1). The faster release rate obtained for the impregnation performed at 20 MPa for 3 h could be explained by the formation of an ibuprofen coating on the polymer’s surface during the depressurization of the system. The same effect has been described elsewhere; however; in that study, the coating was removed prior to ibuprofen quantification [3]. Another explanation may be the structure of the polymer after depressurization. During 3 h at 20 MPa, scCO_2_ has more time to diffuse within the polymer matrix which, after depressurization causes the formation of pores, creating a matrix with a higher porosity [6] therefore facilitating the inward diffusion of buffer in the polymer matrix and ibuprofen outward diffusion into the PBS media.

The release of ibuprofen from the impregnated mcl-PHA matrices was studied over a period of 24 h, in PBS at 37 °C. Figure 5 shows the release of ibuprofen from the samples, which resembles a type II release profile [1]. This is usually referred to as a constant or zero-order release, which allows the drug to be delivered at a constant rate, thus maintaining the API at a stable concentration in the blood stream. This type of release profile is usual for polymers and has become common in commercial systems [1]. This release profile is similar to the one described elsewhere for powder ibuprofen; however, the complete release of ibuprofen is attained after 7 h [38]. In this study we observed that after 4 h the release rate slows down, which also happens in the study by Aroso et al. [38] However, prolonging the study of the release up to 24 h allowed us to conclude that it is possible to have a stable release of ibuprofen during that time period.

## 3. Materials and Methods

### 3.1. mcl-PHA Production and Extraction

The mcl-PHA used in this study was produced and extracted as reported by de Meneses et al. [28]. Briefly, *Pseudomonas chlororaphis* subs. *aurantiaca* (DSM 19603) was grown using glycerol as the sole carbon source in a fed-batch bioreactor cultivation. For the recovery of the mcl-PHA, dried *P. chlororaphis* biomass (10 g) was subjected to Soxhlet extraction with chloroform (270 mL) at 85 °C for 24 h. The obtained solutions were filtered using 0.45 µm filters (Whatman, Maidstone, United Kingdom) and the biopolymer was recovered by precipitation in cold ethanol (1:10, *v*/*v*), under vigorous stirring. The mixture was kept overnight at −20 °C for complete polymer precipitation. The biopolymer was collected and dried in a fume hood at room temperature. It was kept in a closed vessel at room temperature until use.

### 3.2. Characterization

#### 3.2.1. Thermal Properties

Differential scanning calorimetry (DSC) analysis was performed in a DSC Q2000 from TA Instruments Inc. (Tzero DSC technology, New Castle, DE, USA) operating in the Heat Flow T4P option. The measurements were carried out under anhydrous high purity nitrogen at a flow rate of 50 mL/min. DSC Tzero calibration was carried out in the temperature range from −90 to 200 °C. The samples were submitted to two cooling and heating runs between −90 and 120 °C, at a rate of 10 °C/min. The samples were encapsulated in Tzero (aluminium) hermetic pans with a Tzero hermetic lid, without a pinhole to avoid water loss by evaporation.

Thermogravimetric analysis (TGA) was performed with a Labsys EVO (Setaram, Caluire, France). The samples were placed in an aluminum pan and analyzed in a temperature ranging between 25 and 500 °C, using a heating rate of 10 °C/min.

#### 3.2.2. X-ray Diffraction

X-ray diffraction (XRD) analysis was performed using PANalytical’s X’Pert PRO MRD system, with Cu K-alpha radiation over the 2θ range of 10–40°, at a scan rate of 1 deg/min. The crystalline percentage of the polymer was calculated by the ratio of the crystalline area and the total area [46].

#### 3.2.3. Fourier Transform Infrared Spectroscopy

Attenuated total reflection Fourier transform infrared spectroscopy (ATR-FTIR) analysis was conducted in a Perkin Elmer Spectrum Two spectrometer. The mcl-PHA was directly placed in the ATR-FTIR cell and spectra were recorded from 4000 to 400 cm^−1^ resolution with 16 scans at room temperature.

#### 3.2.4. Cytotoxicity

The biopolymer’s biocompatibility was evaluated on an L929 cell line (DSMZ—German Collection of Microorganism and cell culture GmbH), and on an HaCaT cell line (DKFZ, Heidelberg). L929 cells were cultured in Eagle’s Minimum Essential Medium (MEM, with 1.5 g/L sodium bicarbonate, non-essential amino acids, L-glutamine and sodium pyruvate, Corning), supplemented with 10% fetal bovine serum (FBS, Corning) and 1% penicillin-streptomycin (Corning). HaCaT cells were grown in Dulbelco’s Modified Eagle Medium (DMEM with 4.5 g/L glucose, L-glutamine and sodium pyruvate, Corning) supplemented with 10% fetal bovine serum (FBS, Corning) and 1% penicillin-streptomycin (Corning). Both cell lines were cultured in a humidified incubator at 37 °C, with 5% CO_2_.

The cytotoxicity assays were conducted according to ISO/EN 10993-5, for medical devices [33]. PHA extracts (1 mg/mL) were prepared by adding mcl-PHA to MEM or DMEM, for each used cell line, and incubated at 37 °C, for 24 h at 60 rpm. After that period, the media were filtered to remove any particles in suspension. The cell monolayers (1 × 10^4^ cells/well) were incubated with the extracts (2 to 500 ug/mL) for 24 h at 37 °C and 5 % CO_2_. Cells incubated with complete media only were used as the negative control. Cell viability was evaluated using the CellTiter 96^®^ Aqueous One Solution Cell Proliferation Assay (Promega), which is based on tetrazolium active component ((3-(4,5-dimethylthiazol-2-yl)-5-(3-carboxymethoxyphenyl)-2-(4-sulfophenyl)-2H-tetrazolium, MTS). The amount of formazan product was measured in a microplate reader (VICTOR Nivo TM, PerkinElmer, Waltham, MS, USA) at 490 nm, as absorbance is directly proportional to the number of viable cells in culture. Cell viability was expressed as percentage of cells exposed to extracts vs control. Statistical analysis was performed using GraphPad Prism 7.00 software. One-way ANOVA test was performed, as well, as Dunnett’s multiple comparison test. Statistical differences were considered if *p* < 0.05.

### 3.3. Ibuprofen Preparations

Ibuprofen was obtained from ibuprofen sodium salt as described in [38]. A 50 mg/mL solution of ibuprofen sodium salt (98%, Fluka) was prepared in deionized water and acidified with hydrochloric acid (37%, Sigma-Aldrich, Burlington, MA, USA) (3 M) until the pH value was between 1 and 2. Dichloromethane (99.8%, Sigma-Aldrich) (10 mL) was added, and pure ibuprofen was extracted from the organic phase. The extraction procedure was performed four times to ensure that all ibuprofen was recovered. The ibuprofen solution in dichloromethane was left to evaporate completely at room temperature in the fume hood. The obtained white powder (pure ibuprofen) was then weighed and stored at room temperature. The purity of ibuprofen was assessed by ATR-FTIR. This procedure is described to produce ibuprofen with 98% purity [38].

### 3.4. Supercritical CO_2_ Assisted Impregnation

The scCO_2_-assisted impregnation of ibuprofen into the mcl-PHA matrices was performed in a batch apparatus described elsewhere [47]. The mcl-PHA (0.3 g) and ibuprofen (0.3 g) were loaded into the high-pressure vessel (20 mL) without direct contact, and then heated in a bath up to 40 °C. CO_2_ was liquefied in a cooling bath (−15 °C) containing a water/ethylene glycol solution (1:1) and then pumped with a pneumatic metering pump (Williams, V series, Ivyland, PA, USA) to the desired pressure. The pressure inside the vessel was controlled using a pressure transducer. The assays were conducted at 15 or 20 MPa and three impregnation times were tested (30 min, 1 and 3 h). After that period, the system was slowly depressurized, and the samples were recovered. To guarantee complete removal of CO_2_, the samples were left open in a fume hood over-night and weighed until no change in mass was observed. The assays were performed in duplicate. The mcl-PHA ibuprofen- impregnated samples were characterized by FTIR, DSC/TGA and XRD, as described above.

## 3.5. Ibuprofen Release Studies

Ibuprofen release from the impregnated mcl-PHA samples was evaluated through its quantification by spectrophotometric methods. The impregnated samples were immersed in 20 mL of phosphate buffered saline (PBS) (8 g/L NaCl, 0.2 g/L KCl, 1.44 g/L Na_2_HPO_4_, 0.24 g/L KH_2_PO_4_; pH 7.4) and placed in an orbital shaker at 37 °C and 200 rpm. Samples (500 µL) were collected at 0, 5, 10, 15, 30, 45, 60 min, 4, 5 and 24 h. Fresh PBS (500 µL) was added to maintain the buffer’s volume. The absorbance of the samples was measured in a UV/Vis spectrophotometer at 265 nm. A calibration curve was made with an ibuprofen on PBS solution with concentrations ranging from 0.500 to 0.001 mg/mL. PBS was used as zero reference.

The release profile of ibuprofen in PBS was measured as M_t_/M_inf_, where M_t_ represents the mass of ibuprofen released at time t and M_inf_ represents the total mass of ibuprofen released.

The maximum ibuprofen concentration was calculated from Equation (1), where m(ibu)_24_ represents the total mass of ibuprofen released to PBS after 24 h, and m(PHA) represents the mass of mcl-PHA used for the same impregnation assay. The values are presented as average ± standard deviation (SD).

Maximum ibuprofen concentration = m(ibu)_24_/m(PHA) (1)

The ibuprofen impregnation rate at 20 MPa was determined from the slope of the plot of maximum ibuprofen concentration vs. the correspondent impregnation time. The initial ibuprofen release rate (mg/h) was obtained as the slope of the linear phase of ibuprofen release to PBS. The values are presented as average ± SD.

## 4. Conclusions

This work has demonstrated for the first time the preparation of ibuprofen-loaded matrices based on the mcl-PHA produced by the bacterium *Pseudomonas chlororaphis*, a biocompatible natural polyester. Ibuprofen was impregnated into the biopolymer matrix using scCO_2_ as solvent, resulting in a loading of 90.8 mg/g_PHA_ at 20 MPa and at least 1 h of impregnation time. The applied treatment conditions resulted in changes on the polymer thermal properties and crystallinity, with the processed samples displaying lower T_g_, hence suggesting the plasticization of the mcl-PHA. Ibuprofen release from the polymeric matrix followed a type II release profile which is typical for polymeric matrices. Given the adhesive properties of the biopolymer, together with its biocompatibility, the developed structures are promising materials for designing novel topical formulations that allow the delivery of stable concentrations of APIs, such as ibuprofen.

## Figures and Tables

**Table 1:** Impregnation conditions of pressure and time and the obtained ibuprofen impregnation yield on mcl-PHA (mg/g_PHA_), initial ibuprofen release rate to PBS (mg/h) and ibuprofen release during the first hour (%). Results are presented as mean ± SD and experiments were performed in duplicate.

Pressure (MPa)	Time (hours)	Maximum Ibuprofen Concentration (mg/g_PHA_)	Initial Ibuprofen Release Rate (mg/h)	Ibuprofen Release in 1 h (%)
15	3	66.7 ± 0.7	3.7 ± 0.1	21
20	0.5	40.9 ± 0.8	3.0 ± 0.2	32
20	1	90.8 ± 6.5	5.6 ± 0.5	33
20	3	93.3 ± 4.7	14.7 ± 5.2	45

## Data Availability

Not applicable.

## Sample Availability

Samples of the compounds are available upon request from the authors.

## References

[1] Bajpai A.K., Shukla S.K., Bhanu S., Kankane S. (2008). Responsive Polymers in Controlled Drug Delivery. Prog. Polym. Sci..

[2] Barros A.A., Silva J.M., Craveiro R., Paiva A., Reis R.L., Duarte A.R.C. (2017). Green Solvents for Enhanced Impregnation Processes in Biomedicine. Curr. Opin. Green Sustain. Chem..

[3] Champeau M., Thomassin J.M., Tassaing T., Jerome C. (2015). Drug Loading of Sutures by Supercritical CO_2_ Impregnation: Effect of Polymer/Drug Interactions and Thermal Transitions. Macromol. Mater. Eng..

[4] Duarte A.R.C., Mano J.F., Reis R.L. (2009). Supercritical Fluids in Biomedical and Tissue Engineering Applications: A Review. Int. Mater. Rev..

[5] Fleming O.S., Kazarian S.G. (2006). Polymer Processing with Supercritical Fluids. Supercrit. Carbon Dioxide Polym. React. Eng..

[6] Knez Ž.A., Markočič E., Novak Z., Hrnčič M.K. (2011). Processing Polymeric Biomaterials Using Supercritical CO_2_. Chem. Ing. Tech..

[7] Duarte A.R.C., Simplicio A.L., Vega-González A., Subra-Paternault P., Coimbra P., Gil M.H., de Sousa H.C., Duarte C.M.M. (2007). Supercritical Fluid Impregnation of a Biocompatible Polymer for Ophthalmic Drug Delivery. J. Supercrit. Fluids.

[8] Yañez F., Martikainen L., Braga M.E.M., Alvarez-Lorenzo C., Concheiro A., Duarte C.M.M., Gil M.H., De Sousa H.C. (2011). Supercritical Fluid-Assisted Preparation of Imprinted Contact Lenses for Drug Delivery. Acta Biomater..

[9] Barros A.A., Oliveira C., Reis R.L., Lima E., Duarte A.R.C. (2015). Ketoprofen-Eluting Biodegradable Ureteral Stents by CO_2_ Impregnation: In Vitro Study. Int. J. Pharm..

[10] García-González C.A., Jin M., Gerth J., Alvarez-Lorenzo C., Smirnova I. (2015). Polysaccharide-Based Aerogel Microspheres for Oral Drug Delivery. Carbohydr. Polym..

[11] Tang C., Guan Y.X., Yao S.J., Zhu Z.Q. (2014). Preparation of Ibuprofen-Loaded Chitosan Films for Oral Mucosal Drug Delivery Using Supercritical Solution Impregnation. Int. J. Pharm..

[12] Davies O.R., Lewis A.L., Whitaker M.J., Tai H., Shakesheff K.M., Howdle S.M. (2008). Applications of Supercritical CO_2_ in the Fabrication of Polymer Systems for Drug Delivery and Tissue Engineering. Adv. Drug Deliv. Rev..

[13] Banchero M., Mohamed S.S.Y., Leone F., Lopez F., Ronchetti S., Manna L., Onida B. (2019). Supercritical Solvent Impregnation of Different Drugs in Mesoporous Nanostructured Zno. Pharmaceutics.

[14] Campardelli R., Franco P., Reverchon E., De Marco I. (2019). Polycaprolactone/Nimesulide Patches Obtained by a One-Step Supercritical Foaming + Impregnation Process. J. Supercrit. Fluids.

[15] Elmowafy E., Abdal-Hay A., Skouras A., Tiboni M., Casettari L., Guarino V. (2019). Polyhydroxyalkanoate (PHA): Applications in Drug Delivery and Tissue Engineering. Expert Rev. Med. Devices.

[16] Silva J.B., Pereira J.R., Marreiros B.C., Reis M.A.M., Freitas F. (2021). Microbial Production of Medium-Chain Length Polyhydroxyalkanoates. Process Biochem..

[17] Anjum A., Zuber M., Zia K.M., Noreen A., Anjum M.N., Tabasum S. (2016). Microbial Production of Polyhydroxyalkanoates (PHAs) and Its Copolymers: A Review of Recent Advancements. Int. J. Biol. Macromol..

[18] Muhr A., Maria E., Salerno A., Reiterer A., Malli K., Strohmeier K., Schober S., Mittelbach M., Koller M. (2013). Novel Description of Mcl -PHA Biosynthesis by *Pseudomonas chlororaphis* from Animal-Derived Waste. J. Biotechnol..

[19] Pereira J.R., Araújo D., Marques A.C., Neves L.A., Grandfils C., Sevrin C., Alves V.D., Fortunato E., Reis M.A.M.M., Freitas F. (2019). Demonstration of the Adhesive Properties of the Medium-Chain-Length Polyhydroxyalkanoate Produced by *Pseudomonas chlororaphis* Subsp. Aurantiaca from Glycerol. Int. J. Biol. Macromol..

[20] Nigmatullin R., Thomas P., Lukasiewicz B., Puthussery H., Roy I. (2015). Polyhydroxyalkanoates, a Family of Natural Polymers, and Their Applications in Drug Delivery. J. Chem. Technol. Biotechnol..

[21] Lukasiewicz B., Basnett P., Nigmatullin R., Matharu R., Knowles J.C., Roy I. (2018). Binary Polyhydroxyalkanoate Systems for Soft Tissue Engineering. Acta Biomater..

[22] Wang Z., Itoh Y., Hosaka Y., Kobayashi I., Nakano Y., Maeda I., Umeda F., Yamakawa J., Kawase M., Yagi K. (2003). Novel Transdermal Drug Delivery System with Polyhydroxyalkanoate and Starburst Polyamidoamine Dendrimer. J. Biosci. Bioeng..

[23] Gursel I., Yagmurlu F., Korkusuz F., Hasirci V. (2002). In Vitro Antibiotic Release from Poly(3-Hydroxybutyrate-Co-3- Hydroxyvalerate) Rods. J. Microencapsul..

[24] Türesin F., Gürsel I., Hasirci V. (2001). Biodegradable Polyhydroxyalkanoate Implants for Osteomyelitis Therapy: In Vitro Antibiotic Release. J. Biomater. Sci. Polym. Ed..

[25] Antonov E.N., Minaeva S.A., Popov V.K. (2013). A Study of Ibuprofen Solubility in Supercritical Carbon Dioxide by Fourier-Transform Infrared Spectroscopy. Russ. J. Phys. Chem..

[26] Cravo C., Duarte A.R.C., Duarte C.M.M. (2007). Solubility of Carbon Dioxide in a Natural Biodegradable Polymer: Determination of Diffusion Coefficients. J. Supercrit. Fluids.

[27] Takahashi S., Hassler J.C., Kiran E. (2012). Melting Behavior of Biodegradable Polyesters in Carbon Dioxide at High Pressures. J. Supercrit. Fluids.

[28] De Meneses L., Pereira J.R., Sevrin C., Grandfils C., Paiva A., Reis M.A.M., Freitas F. (2020). *Pseudomonas chlororaphis* as a Multiproduct Platform: Conversion of Glycerol into High-Value Biopolymers and Phenazines. N. Biotechnol..

[29] Sathiyanarayanan G., Bhatia S.K., Song H.S., Jeon J.M., Kim J., Lee Y.K., Kim Y.G., Yang Y.H. (2017). Production and Characterization of Medium-Chain-Length Polyhydroxyalkanoate Copolymer from Arctic Psychrotrophic Bacterium *Pseudomonas* Sp. PAMC 28620. Int. J. Biol. Macromol..

[30] Tanikkul P., Sullivan G.L., Sarp S., Pisutpaisal N. (2020). Biosynthesis of Medium Chain Length Polyhydroxyalkanoates (Mcl-PHAs) from Palm Oil. Case Stud. Chem. Environ. Eng..

[31] Rebocho A.T., Pereira J.R., Neves L.A., Alves V.D., Sevrin C., Grandfils C., Freitas F., Reis M.A.M. (2020). Preparation and Characterization of Films Based on a Natural p(3hb)/Mcl-Pha Blend Obtained through the Co-Culture of *Cupriavidus necator* and *Pseudomonas citronellolis* in Apple Pulp Waste. Bioengineering.

[32] López-Cuellar M.R., Alba-Flores J., Rodríguez J.N.G., Pérez-Guevara F. (2011). Production of Polyhydroxyalkanoates (PHAs) with Canola Oil as Carbon Source. Int. J. Biol. Macromol..

[33] The International Organization for Standardization (2009). Biological Evaluation of Medical Devices—Part 5: Tests for In Vitro Cytotoxicity.

[34] Panaitescu D.M., Lupescu I., Frone A.N., Chiulan I., Nicolae C.A., Tofan V., Stefaniu A., Somoghi R., Trusca R. (2017). Medium Chain-Length Polyhydroxyalkanoate Copolymer Modified by Bacterial Cellulose for Medical Devices. Biomacromolecules.

[35] Ji Y., Li X.T., Chen G.Q. (2008). Interactions between a Poly(3-Hydroxybutyrate-Co-3-Hydroxyvalerate-Co-3-Hydroxyhexanoate) Terpolyester and Human Keratinocytes. Biomaterials.

[36] Sun J., Dai Z., Zhao Y., Chen G.Q. (2007). In Vitro Effect of Oligo-Hydroxyalkanoates on the Growth of Mouse Fibroblast Cell Line L929. Biomaterials.

[37] Bagheri H., Ghader S., Hatami N. (2019). Solubility of Ibuprofen in Conventional Solvents and Supercritical CO_2_: Evaluation of Ideal and Non-Ideal Models. Chem. Chem. Technol..

[38] Aroso I.M., Craveiro R., Rocha Â., Dionísio M., Barreiros S., Reis R.L., Paiva A., Duarte A.R.C. (2015). Design of Controlled Release Systems for THEDES—Therapeutic Deep Eutectic Solvents, Using Supercritical Fluid Technology. Int. J. Pharm..

[39] Cooper A.I. (2003). Porous Materials and Supercritical Fluids. Adv. Mater..

[40] Martins M., Aroso I.M., Reis R.L., Duarte A.R.C., Craveiro R., Paiva A. (2014). Enhanced Performance of Supercritical Fluid Foaming of Natural-Based Polymers by Deep Eutectic Solvents. AIChE J..

[41] Nikitin L.N., Gallyamov M.O., Vinokur R.A., Nikolaec A.Y., Said-Galiyev E.E., Khokhlov A.R., Jespersen H.T., Schaumburg K. (2003). Swelling and Impregnation of Polystyrene Using Supercritical Carbon Dioxide. J. Supercrit. Fluids.

[42] Ramukutty S., Ramachandran E. (2012). Growth, Spectral and Thermal Studies of Ibuprofen Crystals. Cryst. Res. Technol..

[43] Yoganathan R., Mammucari R., Foster N.R. (2010). Impregnation of Ibuprofen into Polycaprolactone Using Supercritical Carbon Dioxide. J. Phys. Conf. Ser..

[44] Bidone J., Melo A.P.P., Bazzo G.C., Carmignan F., Soldi M.S., Pires A.T.N., Lemos-Senna E. (2009). Preparation and Characterization of Ibuprofen-Loaded Microspheres Consisting of Poly(3-Hydroxybutyrate) and Methoxy Poly (Ethylene Glycol)-b-Poly (D,L-Lactide) Blends or Poly(3-Hydroxybutyrate) and Gelatin Composites for Controlled Drug Release. Mater. Sci. Eng. C.

[45] Mallick S., Pattnaik S., Swain K., De P.K., Saha A., Mazumdar P., Ghoshal G. (2008). Physicochemical Characterization of Interaction of Ibuprofen by Solid-State Milling with Aluminum Hydroxide. Drug Dev. Ind. Pharm..

[46] Galego N., Rozsa C., Sánchez R., Fung J., Analía V., Santo Tomás J. (2000). Characterization and Application of Poly(B-Hydroxyalkanoates) Family as Composite Biomaterials. Polym. Test..

[47] Martins M., Craveiro R., Paiva A., Duarte A.R.C., Reis R.L. (2014). Supercritical Fluid Processing of Natural Based Polymers Doped with Ionic Liquids. Chem. Eng. J..

